# Pathological Investigation of Congenital Bicuspid Aortic Valve Stenosis, Compared with Atherosclerotic Tricuspid Aortic Valve Stenosis and Congenital Bicuspid Aortic Valve Regurgitation

**DOI:** 10.1371/journal.pone.0160208

**Published:** 2016-08-01

**Authors:** Yasuhiro Hamatani, Hatsue Ishibashi-Ueda, Toshiyuki Nagai, Yasuo Sugano, Hideaki Kanzaki, Satoshi Yasuda, Tomoyuki Fujita, Junjiro Kobayashi, Toshihisa Anzai

**Affiliations:** 1 Department of Cardiovascular Medicine, National Cerebral and Cardiovascular Center, Osaka, Japan; 2 Department of Pathology, National Cerebral and Cardiovascular Center, Osaka, Japan; 3 Department of Cardiovascular Surgery, National Cerebral and Cardiovascular Center, Osaka, Japan; Cincinnati Children's Medical Center, UNITED STATES

## Abstract

**Background:**

Congenital bicuspid aortic valve (CBAV) is the main cause of aortic stenosis (AS) in young adults. However, the histopathological features of AS in patients with CBAV have not been fully investigated.

**Methods and Results:**

We examined specimens of aortic valve leaflets obtained from patients who had undergone aortic valve re/placement at our institution for severe AS with CBAV (n = 24, CBAV-AS group), severe AS with tricuspid aortic valve (n = 24, TAV-AS group), and severe aortic regurgitation (AR) with CBAV (n = 24, CBAV-AR group). We compared the histopathological features among the three groups. Pathological features were classified using semi-quantitative methods (graded on a scale 0 to 3) by experienced pathologists without knowledge of the patients’ backgrounds. The severity of inflammation, neovascularization, and calcium and cholesterol deposition did not differ between the CBAV-AS and TAV-AS groups, and these four parameters were less marked in the CBAV-AR group than in the CBAV-AS (all p<0.01). Meanwhile, the grade of valvular fibrosis was greater in the CBAV-AS group, compared with the TAV-AS and CBAV-AR groups (both p<0.01). In AS patients, thickness of fibrotic lesions was greater on the aortic side than on the ventricular side (both p<0.01). Meanwhile, thickness of fibrotic lesions was comparable between the aortic and ventricular sides in CBAV-AR patients (p = 0.35).

**Conclusions:**

Valvular fibrosis, especially on the aortic side, was greater in patients with CBAV-AS than in those without, suggesting a difference in the pathogenesis of AS between CBAV and TAV.

## Introduction

Congenital bicuspid aortic valve (CBAV) is a common congenital heart malformation, with an estimated prevalence between 0.5 and 2% [1, 2]. CBAV has been identified as the main cause of aortic stenosis (AS) requiring surgical treatment in children and young adults. Despite its prevalence, the pathophysiology of AS in CBAV remains unclear [3].

In histological studies, stenotic aortic valve and atherosclerosis share several common features, including lipid accumulation, calcification, infiltration of inflammatory cells and neovascularization [4, 5]. The progression of AS in the tricuspid aortic valve (TAV) is associated with traditional atherosclerotic risk factors [6, 7], and AS in TAV might result from an active process similar to atherosclerosis. To date, few data exist to explain the mechanisms of the development of AS in patients with CBAV. Although atherosclerotic risk factors are also reported to be associated with increased risk of AS in patients with CBAV [8], the precise mechanisms and histopathological features of AS in patients with CBAV have not been fully elucidated.

In addition, CBAV is often associated with abnormalities of the ascending aortic media, resulting in aortic bulb dilatation and aortic regurgitation (AR). Patients with AR develop symptoms and undergo aortic valve replacement at a younger age compared with those with AS. Histological comparison of AS and AR in CBAV patients could clarify the time course and mechanisms of the development of AS in CBAV.

The aim of this study was to investigate the histopathological characteristics of AS in patients with CBAV, compared with AS patients with TAV and AR patients with CBAV.

## Materials and Methods

### Study population and data collection

We examined specimens of aortic valve leaflets obtained from patients who had undergone aortic valve replacement from April 2010 to September 2015 at our institution for severe AS with CBAV (n = 24, CBAV-AS group). Groups comprising 24 patients with severely stenotic TAV (TAV-AS group) and 24 CBAV patients with severe AR (CBAV-AR group) were enrolled in parallel. We compared patients’ backgrounds and pathological characteristics among patients with CBAV-AS, TAV-AS and CBAV-AR.

Valve tissue was collected at the time of the operation. Patients’ medical records were reviewed to assess the clinical data. This study conformed to the principles outlined in the Declaration of Helsinki. In this study, because patients’ information was anonymized and de-identified prior to analyses, written informed consent was not obtained from each patient. However, we publicized the study by posting a summary of the protocol on the website of the National Cerebral and Cardiovascular Center, and clearly informed patients of their right to refuse enrollment. The full study including these procedures for informed consent and enrollment were approved by the Institutional Review Board of the National Cerebral and Cardiovascular Center (M27-076).

### Definition of measurements

We reviewed clinical data in the patients’ medical charts. Hypertension was diagnosed if peripheral blood pressure was >140/90 mmHg or if the patient was taking medication for hypertension. Diabetes mellitus was defined as HbA1c ≥6.5% or receiving anti-diabetic medication. Dyslipidemia was diagnosed if total cholesterol was >220 mg/dL, if low-density lipoprotein cholesterol was >140 mg/dL, if triglyceride was >150 mg/dL, if high-density lipoprotein cholesterol was <40 mg/dL, or if the patient was taking a lipid-lowering agent. All patients underwent cardiac catheterization or coronary computed tomography angiography before operation. Coronary artery disease was defined as coronary stenosis of at least 75% (in case of the left main trunk, the cut off was ≥ 50%) detected by coronary angiography before the operation. All patients also underwent trans-thoracic echocardiography before operation. Severe AS and severe AR were defined according to the guidelines [9, 10]. Severe AR patients with moderate or severe AS were excluded in this analysis. We also excluded patients complicated with infective endocarditis.

### Pathological analysis

The valve samples were obtained vertically through the valve cusps near the center of each leaflet. The aortic valve was fixed in 10% buffered formalin and embedded in paraffin using standard histological procedures. Representative lesions of the obtained materials were macroscopically selected for further processing. The paraffin-embedded specimens were sectioned at 4–5 μm thickness, and stained with hematoxylin-eosin (H&E) and Masson’s trichrome (MT) after decalcification with 10% EDTA solution. We assessed the following factors; neovascularization, inflammation, calcification/cholesterol deposition, and valvular fibrosis. We also measured the whole layer thickness of the valve and the thickness of fibrotic lesion (measured on the aortic side and ventricular side) at the mid-portion of the leaflet.

Immunohistochemical examinations were performed on 4-5-μm-thick 10% buffered formalin-fixed and paraffin-embedded tissue sections, using monoclonal antibodies to confirm inflammation, neovascularization, calcification, and extracellular matrix deposit besides H&E and MT stainings. All steps were performed using an auto-immunostainer, Bond-III (Leica, Japan), according to the manufacturer’s instructions. All slides were incubated with primary monoclonal antibodies against CD3 for T cells (Dako, Denmark) for 15 minutes. Representative samples were additionally incubated with primary antibodies against von Willebrand factor (Dako, Japan) for endothelial cells (neovascularization), osteopontin (Leica, Japan) for calcium deposits, and tenascin-C (4C-8, IBL, Japan), as the extracellular matrix which was reportedly associated with progression of AS [11], followed by incubation with a mouse-rabbit-horseradish peroxidase polymer and 3,3’- diaminobenzidine substrate. The sections were then incubated in primer (anti-rabbit and anti-mouse) for 5 minutes. The primary antibody was omitted from these protocols as a negative control. The sections were subsequently counterstained with hematoxylin-eosin.

### Scoring of immunohistochemical and pathological features

Immunohistochemical and histopathological features were classified using semi-quantitative methods (graded on a scale 0 to 3) by experienced pathologists without knowledge of the patients’ clinical data. In terms of the semi-quantitative scoring of immunohistochemical staining, positive cells (for T cells)/area were classified as follows; grade 0 was no positive cells/area, grade 1 was <25% cells positive/area, grade 2 was 25–50% cells positive/area, and grade 3 was > 50% cells positive/area [12]. The scoring for neovascularization was classified as follows: grade 0, absence; grade 1, isolated neovessels; grade 2, minimal aggregates; grade 3, abundant neovessels [13].

Calcification was also graded on a scale of 0 to 3, as previously described. Briefly, grade 0 valves showed no calcification, grade 1 valves showed early calcific nodules, grade 2 valves showed several calcific nodules with mild structural distortion, and grade 3 valves showed many several calcific nodules with severe structural distortion [14, 15]. Valvular fibrosis was graded as follows; grade 0: absent or minimal intensity of fibrotic tissue (representing about less than 1% of the section), grade 1: slight intensity of fibrotic tissue (representing about between 1 and 25% of the section), grade 2: moderate intensity of fibrotic tissue (representing about between 25 and 50% of the section), grade 3: severe intensity of fibrotic tissue (representing about more than 50% of the section) according to a previous study [16].

### Statistical analysis

Continuous variables are expressed as mean ± standard deviation. Categorical variables are presented as numbers and percentages. Categorical variables were compared using the chi-squared test when appropriate; otherwise, Fisher’s exact test was used. Continuous variables were compared among the three groups using one-way analysis of variance. Histopathological scores were analyzed using the Wilcoxon rank sum test. Bonferroni correction was used to adjust for multiple comparison. Comparisons within groups were performed using paired t-test. Multivariate linear regression modeling was used to adjust for suspected confounders. Relevant covariates were selected for inclusion in multivariate models based on prior clinical knowledge. JMP version 10 (SAS Institute, Cary, NC) was used to perform all analyses. Two-sided p values less than 0.05 were considered statistically significant.

## Results

### Patients’ characteristics

A total of 24 patients with TAV-AS, 24 patients with CBAV-AS, and 24 patients with CBAV-AR were included in this study. Clinical characteristics, co-morbid conditions, echocardiographic parameters, and laboratory data in the three groups are shown in Table 1. There were some differences in baseline characteristics among the three groups. Patients with CBAV-AR were more often male, younger, and had lower left ventricular ejection fraction (all p < 0.01). Co-morbid conditions such as hypertension, diabetes mellitus, dyslipidemia, and coronary artery disease were most prevalent in patients with TAV-AS (all p < 0.01).

**Table 1:** Baseline characteristics of patients in three groups.

	TAV-AS	CBAV-AS	CBAV-AR	p value
Number	24	24	24	
**Baseline characteristics**				
Male	12 (50%)	14 (58%)	23 (96%)	<0.01
Age (years)	77 ± 6	62 ± 13	39 ± 13	<0.01
Smoking history	9 (38%)	6 (25%)	8 (33%)	0.71
**Co-morbid conditions**				
Hypertension	22 (92%)	9 (38%)	5 (20%)	<0.01
Diabetes mellitus	9 (38%)	4 (17%)	0 (0%)	<0.01
Dyslipidemia	20 (83%)	12 (50%)	7 (29%)	<0.01
Coronary artery disease	10 (42%)	0 (0%)	0 (0%)	<0.01
Ascending aorta diameter (mm)	33 ± 3	41 ± 5	38 ± 7	<0.01
Ascending aorta dilatation (≥45 mm)	0 (0%)	5 (21%)	3 (14%)	0.07
**Echocardiographic parameters**				
LVEF (%)	61 ± 8	62 ± 7	52 ± 14	0.01
AVA (cm^2^)	0.72 ± 0.16	0.77 ± 0.26	-	0.37
Mean PG (mmHg)	47 ± 14	62 ± 26	-	0.02
**Laboratory data**				
Estimated GFR (ml/m^2^)	52.3 ± 16.1	67.5 ± 9.2	69.4 ± 13.1	0.01
Blood urea nitrogen (mg/dl)	20 ± 8	16 ± 4	16 ± 6	0.01
Creatinine (mg/dl)	1.0 ± 0.6	0.8 ± 0.2	0.9 ± 0.2	0.16
HbA1c (%)	5.9 ± 0.8	5.7 ± 0.5	5.4 ± 0.4	0.01
Total cholesterol (mg/dl)	174 ± 41	185 ± 25	182 ± 29	0.47
LDL cholesterol (mg/dl)	97 ± 21	105 ± 17	108 ± 21	0.39
HDL cholesterol (mg/dl)	51 ± 19	55 ± 13	50 ± 14	0.57
Triglyceride (mg/dl)	115 ± 59	109 ± 48	136 ± 61	0.55

Categorical data are presented as number (%). Continuous data are presented as mean ± standard deviation. TAV; tricuspid aortic valve, AS; aortic stenosis, CBAV; congenital bicuspid aortic valve, AR; aortic regurgitation, LVEF; left ventricular ejection fraction, AVA; aortic valve area, PG; pressure gradient, GFR; glomerular filtration ratio, HbA1c; hemoglobin A1c, LDL; low density lipoprotein, HDL; high density lipoprotein.

### Histological findings

**Fig 1:**
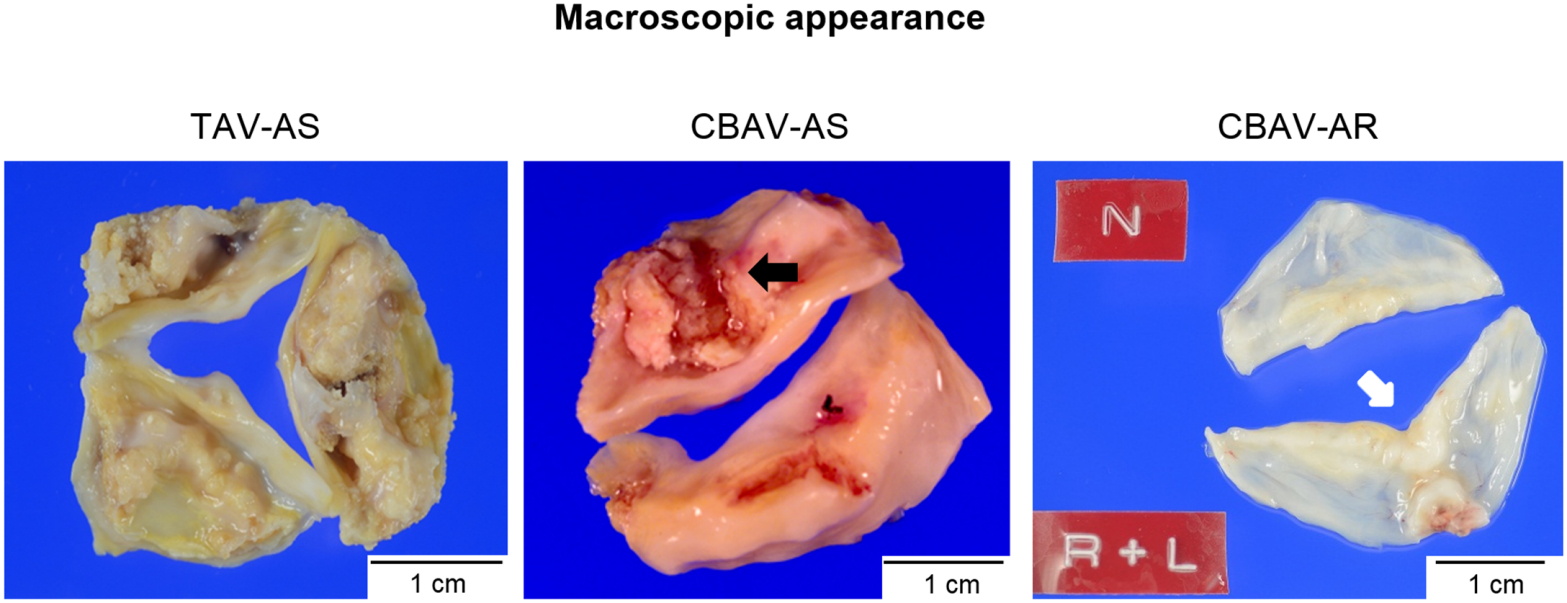
Macroscopic appearances of representative excised aortic valves for aortic stenosis and regurgitation. Tricuspid aortic valve stenosis (TAV-AS); Severe calcified sclerotic tricuspid valve with fused commissures is seen. Congenital bicuspid aortic valve stenosis (CBAV-AS); Two calcified cusps with raphe (black arrow) and severe fibrous thickening are seen. Congenital bicuspid aortic valve regurgitation (CBAV-AR); R (right coronary cusp) and L (left coronary cusp) are fused. Coaptation sites are rather thicker than the other portions (white arrow).

**Fig 2:**
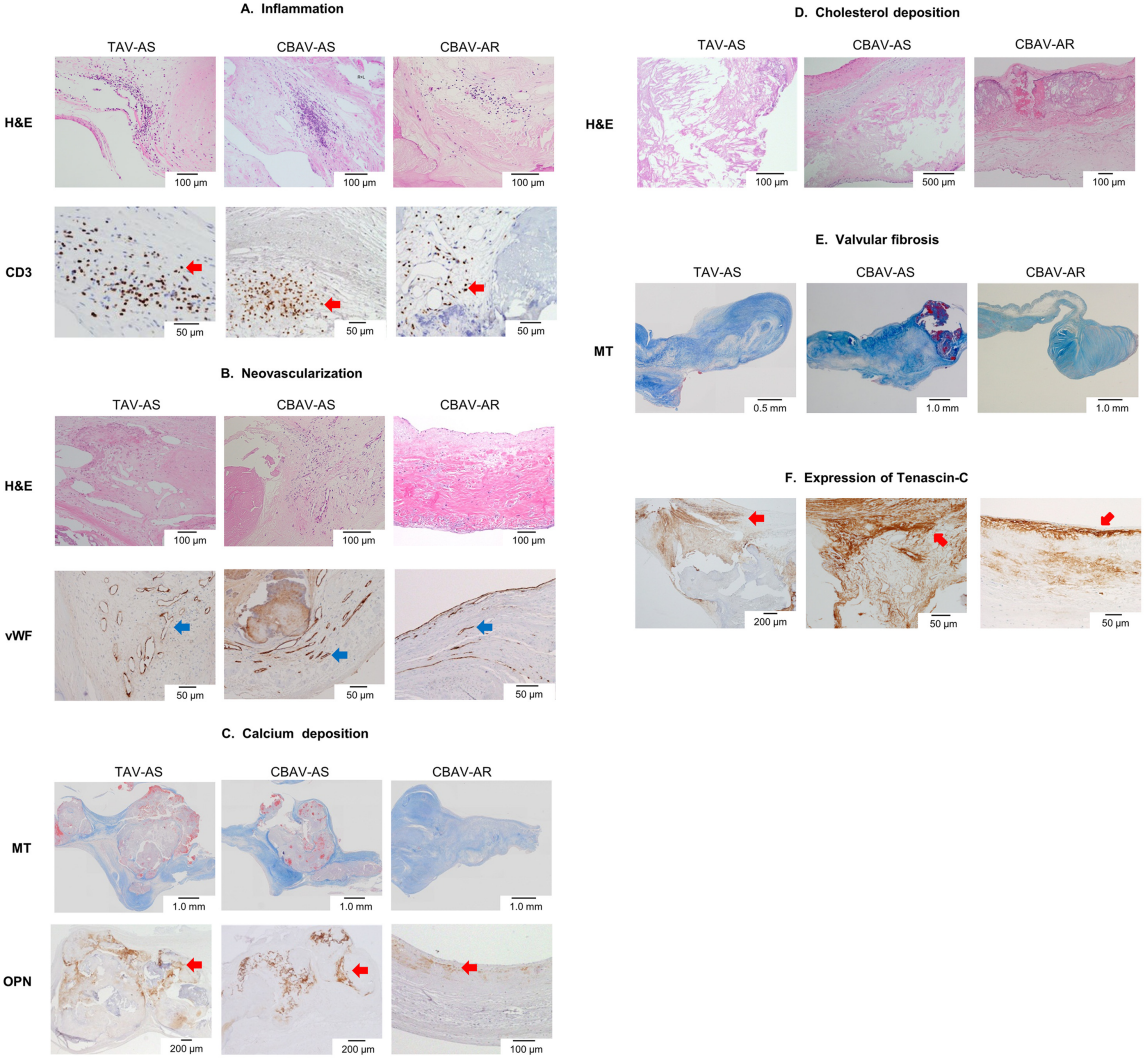
Histological and immunohistochemical findings of aortic valve in TAV-AS, CBAV-AS, and CBAV-AR. A: Inflammation; Inflammation with CD3 is seen in each group. More inflammatory cell infiltration is noted in TAV-AS and CBAV-AS compared with CBAV-AR. Red arrows indicate T cells infiltrations in valves. Upper photomicrographs are hematoxylin-eosin (H&E) staining, and lower photomicrographs are immunohistochemical staining for CD3. B: Neovascularization; Neovascularizations in TAV-AS and CBAV-AS are more intensive than those in CBAV-AR. Upper photomicrographs are H&E staining, and lower photomicrographs are immunohistochemical staining for von Willebrand factor (vWF). Blue arrows indicate the expression of vWF. C: Calcium deposition; The degrees of calcium deposition are higher in TAV-AS and CBAV-AS compared with CBAV-AR. Upper photomicrographs are Masson’s trichrome (MT) staining, and lower photomicrographs are immunohistochemical staining for osteopontin (OPN). Red arrows indicate the expression of osteopontin. D: Cholesterol deposition; The degrees of cholesterol deposition are higher in TAV-AS and CBAV-AS compared with CBAV-AR. Photomicrographs are H&E staining. E: Fibrosis; Valvular fibrosis is more severely noted in CBAV-AS compared with TAV-AS and CBAV-AR. Photomicrographs are MT staining. F: Immunohistochemical staining for tenascin-C. Red arrows indicate the expression of tenascin-C. Tenascin-C deposit is more diffuse and intensive in CBAV-AS valve.

Representative macroscopic images are shown in Fig 1. The severities of inflammation, neovascularization, calcium deposition, cholesterol deposition, and fibrosis are shown in Table 2. In patients with CBAV, the severity of the inflammation, neovascularization, calcium deposition, and cholesterol deposition was higher in patients with CBAV-AS than in those with CBAV-AR (all p < 0.01). Meanwhile, in patients with AS, the severity of inflammation, neovascularization, calcium deposition, and cholesterol deposition did not differ between patients with CBAV-AS and those with TAV-AS (Table 2, Fig 2).

**Table 2:** Histopathological differences between the three groups.

	TAV-AS	CBAV-AS	CBAV-AR	p value between TAV-AS vs. CBAV-AS	p value between CBAV-AS vs. CBAV-AR
Number	24	24	24		
**Inflammation**					
Grade 0	4	3	17	NS	<0.01
Grade 1	16	18	7		
Grade 2	4	3	0		
Grade 3	0	0	0		
**Neovascularization**					
Grade 0	4	1	12	NS	<0.01
Grade 1	13	19	11		
Grade 2	7	4	1		
Grade 3	0	0	0		
**Calcium deposit**					
Grade 0	0	0	15	NS	<0.01
Grade 1	0	0	7		
Grade 2	3	4	2		
Grade 3	21	20	0		
**Cholesterol deposit**					
Grade 0	5	8	18	NS	<0.01
Grade 1	12	8	4		
Grade 2	6	6	2		
Grade 3	1	2	0		
**Fibrosis**					
Grade 0	0	0	0	<0.01	<0.01
Grade 1	18	1	9		
Grade 2	6	9	12		
Grade 3	0	14	3		

TAV; tricuspid aortic valve, CBAV; congenital bicuspid aortic valve, AS; aortic stenosis, AR; aortic regurgitation, NS; not significant.

Valvular fibrosis was different among the three groups. Patients with CBAV-AS had more marked valvular fibrosis than those with CBAV-AR (p < 0.01). In AS patients, valvular fibrosis was more severe in patients with CBAV-AS compared with those with TAV-AS (p < 0.01, Table 2, Fig 2). Intense tenascin-C deposition in the valve was observed in patients with CBAV-AS (Fig 2).

Bicuspid morphology was independently associated with valvular fibrosis even after adjustment for confounders such as age, sex, hypertension, diabetes mellitus, dyslipidemia, and smoking history in multivariate linear regression analysis in AS patients (p < 0.01, Table 3).

**Table 3:** Multivariate linear regression analysis of aortic valvular fibrosis in patients with severe AS.

	Valvular fibrosis	
	Standardized coefficient	p value
Bicuspid morphology	0.84	<0.01
Age (/1 years)	0.02	0.91
Female sex	-0.12	0.31
Hypertension	0.06	0.61
Diabetes mellitus	0.02	0.89
Dyslipidemia	-0.07	0.52
Smoking history	0.09	0.41

AS; aortic stenosis

### Valvular thickness and thickness of fibrotic lesions in patients with CBAV and TAV

Valvular thickness of whole zone and thickness of fibrotic lesions measured on the aortic side and ventricular side are shown in Table 4. Valvular thickness was greatest in patients with CBAV-AS and was thinnest in patients with CBAV-AR. Thickness of fibrotic lesions was also greater in patients with CBAV-AS, compared with TAV-AS patients and CBAV-AR patients (p < 0.01). In AS patients, valvular fibrosis was larger on the aortic side than on the ventricular side (both p < 0.01). Meanwhile, valvular fibrosis was comparable between the aortic and ventricular sides in CBAV-AR patients (p = 0.35).

**Table 4:** Comparison of thickness of valve and fibrotic lesions.

	TAV-AS	CBAV-AS	CBAV-AR	p value
Number	24	24	24	
Thickness of valve (mm)	3.62 ± 1.31	5.39 ± 1.96	0.90 ± 0.25	<0.01
Thickness of fibrotic lesions				
Aortic side (mm)	0.54 ± 0.16	1.23 ± 0.65	0.34 ± 0.13	<0.01
Ventricular side (mm)	0.20 ± 0.12	0.42 ± 0.21	0.29 ± 0.15	<0.01

Continuous data are presented as mean ± standard deviation. TAV; tricuspid aortic valve, AS; aortic stenosis, CBAV; congenital bicuspid aortic valve, AR; aortic regurgitation

## Discussion

The major findings of this study were as follows: First, the severity of inflammation and neovascularization was comparable between patients with CBAV-AS and those with TAV-AS, and was less in patients with CBAV-AR. Second, fibrosis was more prominent in patients with CBAV-AS than in those with TAV-AS and those with CBAV-AR. Third, the valvular fibrosis was greater on the aortic side than on the ventricular side in AS patients, and it was greater in patients with CBAV-AS than in those with CBAV-AR and TAV-AS. These findings suggest that an inappropriate fibrotic response to chronic inflammation and neovascularization, especially on the aortic side, may contribute to the development of AS in CBAV.

### Inflammation and neovascularization in CBAV-AS and TAV-AS

Previous reports suggested that CBAV-AS was associated with increased inflammation and neovascularization compared with TAV-AS [14, 17, 18]. Increased shear stress, genetic mutation, and intraleaflet hemorrhage are reported to accelerate inflammatory processes in CBAV-AS; however, this remains speculative. In our study, the degree of inflammation and neovascularization were comparable between CBAV-AS and TAV-AS. Although differences in the patients’ background, such as age, disease duration and severity of AS, might have caused the discrepant results, the fibrotic response to inflammation rather than inflammation itself could be related to the development of AS in CBAV. Further studies are needed to investigate whether accelerated inflammation and neovascularization truly exist and play a role in the rapid progression of stenosis in patients with CBAV.

### Valvular fibrosis in stenotic bicuspid aortic valve

In our study, valvular fibrosis and thickness of fibrotic lesions were greater in patients with CBAV-AS than in those with TAV-AS, despite no differences in other pathologic parameters. Bicuspid morphology was an independent predictor of valvular fibrosis in patients with AS. In addition, valvular fibrosis was more marked in patients with CBAV-AS than in patients with CBAV-AR. The pathological features of CBAV-AR might reflect the initial lesions prior to the development of CBAV-AS, since CBAV-AR usually occurs in younger patients. Our study suggested that the increased fibrosis in CBAV-AS might be an acquired response.

There are possible explanations for the association between valvular fibrosis and CBAV-AS. First, perturbation of blood flow in CBAV causes valvular thickening and fibrosis. Although the mechanisms by which mechanical forces invoke cellular and molecular responses within the aortic valve are not well understood, cardiovascular tissue generally responds to increased radial stress by increasing in thickness to buffer or neutralize mechanical stress [19]. Second, the valvular fibrosis and progression of AS in CBAV might be related to impairment of the nitric oxide system. A previous study using a porcine model suggested that nitric oxide inhibits calcification processes in aortic valve cells [20]. In humans with CBAV, expression of endothelial nitric oxide synthase (eNOS) in aortic endothelial cells has been reported to be significantly reduced [21]. In fact, eNOS-deficient mice are commonly complicated with CBAV [22, 23]. Moreover, a study using eNOS knock out mice showed the development of aortic valvular fibrosis and calcification in CBAVs, suggesting that nitric oxide deficiency might cause aortic valve sclerosis by promoting fibrosis [24]. Therefore, it is possible that abnormality of nitric oxide synthesis in patients with CBAV might be involved in the pathogenesis of CBAV-AS.

In terms of the thickness of fibrosis, both patients with CBAV-AS and TAV-AS had thicker fibrosis on the aortic side than on the ventricular side. Calcification of the aortic valve usually begins on the aortic side of valve cusps, with relative sparing of the ventricular side. The aortic side of valve cusps is exposed to high mechanical stress of downstream flow during the diastolic phase. In addition, expression of inhibitors of osteogenic signaling is significantly reduced in endothelium from the aortic side of valve cusps [25, 26], leading to dominant calcific change on the aortic side of valve cusps. Although it remains unknown whether mechanical stress and/or a molecular mechanism causes fibrosis in AS patients, our study revealed that fibrosis was also dominant on the aortic side of valve cusps.

In the present study, tenascin-C was markedly expressed in the aortic valve of patients with CBAV-AS. Recent reports have suggested that tenascin-C is expressed in association with the development of cardiovascular diseases, and it may accelerate or sustain fibrosis in cardiovascular tissues [27–30]. The result of intense deposition of tenascin-C supports the idea that inappropriate valvular fibrosis may be the main cause of the development of CBAV-AS. A further study with a larger sample size is warranted.

### Study Limitations

Our study had several limitations. First, the number of samples in the present study was too small to possess sufficient statistical power. Second, the degree of the histological findings was only classified using semi-quantitative methods. Third, in this study, we examined specimens with advanced stage requiring aortic valve surgery for severe stenosis and/or regurgitation. We did not investigate normal aortic valves as controls, and we did not obtain specimens of valves with an earlier stage of stenosis and/or regurgitation. Moreover, less valve degeneration occurs in the settings of aortic root dilatation or prolapse of the cusps, which are the main causes of aortic regurgitation [31]. Therefore, we could not definitively address the causes and mechanisms of stenosis and regurgitation of the aortic valve in cases of CBAV and/or TAV. Fourth, we assessed the cholesterol deposition by H&E staining because the snap frozen section for more specific staining such as Oil Red O was difficult to assess due to valve calcification.

### Conclusion

Inflammation, neovascularization, cholesterol deposition, and calcium deposition were more prominent in patients with CBAV-AS than in those with CBAV-AR, but not significantly different between patients with CBAV-AS and those with TAV-AS. Meanwhile, fibrosis of the aortic valve, especially on the aortic side, was significantly more severe in patients with CBAV-AS than in those without, suggesting a difference in the pathogenesis of AS between CBAV and TAV.
